# Loneliness and problematic internet use: testing the role of interpersonal problems and motivation for internet use

**DOI:** 10.1186/s12888-021-03457-y

**Published:** 2021-09-10

**Authors:** Nahathai Wongpakaran, Tinakon Wongpakaran, Manee Pinyopornpanish, Sutapat Simcharoen, Pimolpun Kuntawong

**Affiliations:** 1 Department of Psychiatry, Faculty of Medicine, Chiang Mai University, 110 Intawaroros Rd., T. Sriphum, A. Muang, Chiang Mai, 50200 Thailand; 2 Jittavej Nakhon Sawan Ratchanakarin Hospital, Nakhon Sawan, Thailand

**Keywords:** Loneliness, Interpersonal problems, Internet addiction, Mediation, Moderation, Structural equation model

## Abstract

**Background:**

A number of factors have been demonstrated to be associated with Problematic Internet Use (PIU); otherwise known as Internet Addiction), which is mostly concerned with psychological problems such as loneliness. This study aimed to examine how and in what way loneliness influenced PIU.

**Methods:**

A self-report measurement on loneliness, the Internet addiction test (IAT) and instruments on interpersonal problems were administered to 318 medical students (57% females); mean age totaled 20.88 years (SD = 1.8). We performed a mediation analysis to evaluate direct effects of loneliness on IAT, as well as indirect effects mediated by interpersonal problems. In addition, motivation for internet use was added to the mediation model and tested whether it acted as the second mediator (serial mediation model) or a moderator (moderated mediation model).

**Results:**

After controlling for sex and age, socially inhibited problems exhibited full mediation whereas the remainder showed partial mediation effects, with the exception that intrusive and cold interpersonal problems indicated no mediating role. Negative motivation and motivation for being accepted had mediation effects for all types of interpersonal problems. Motivation for working was found to be a significant mediator and moderator of the most interpersonal problems. Intrusive and cold styles became a mediator only when some motivation variables were added to the model, implying that not only psychological problems should be included when analyzing PIU, but also other variables such motivation for internet use. The percent of variance explained, by IAT score, increased from 13% in the mediation model to 33% by the moderated mediation model, and 43% using the serial mediation model.

**Conclusion:**

The study suggested the crucial role of loneliness and interpersonal problems on PIU, for which motivation for internet use explained how each interpersonal problem would be associated. This may provide some insight regarding the pathological characteristics of those using the internet as a coping strategy.

**Supplementary Information:**

The online version contains supplementary material available at 10.1186/s12888-021-03457-y.

## Background

Problematic Internet Use (PIU), otherwise known as Internet Addiction, refers to a generalized impulse control disorder involving the problems one experiences in regulating the desire to engage in online activities [1]. According to Young, Internet addiction could compare its symptoms with those of pathological gambling as defined in the DSM-IV [2]. PIU could be considered a mental disorder requiring professional treatment when the individual exhibits significant levels of impairment. Traditionally, research on PIU has focused on direct effects models investigating the associations between psychological vulnerabilities and PIU. Psychological vulnerabilities that have been explored included depression [3], low self-esteem [4], loneliness and shyness [5, 6].

In addition to psychological vulnerability, preconditions associated with developing behavior addictions were found to predict PIU. Such conditions include personality trait of high sensation-seeking [7], insecure attachment [8], and problematic interpersonal styles [9]. As we have already known, the cause of PIU is rather complex [3]. Loneliness, a common state of emotion occurring across lifespan [10], is linked to PIU among adolescents or young adults [11–14]. Loneliness can have either a direct or indirect effect on PIU. Researchers have found that it could be mediated by shyness, family support, self-esteem, social anxiety, depression and interpersonal problems in rendering IA [5, 6, 15–20]. PIU is also related to personality traits [21], while personality traits are related to interpersonal relationship and problems [22–26], and all are related to loneliness. Interpersonal problems were the authors’ subject of interest because they were more similar to personality traits [27] but more sensitive to change. Thus, these problems are considered possible modifiable variables [27–30].

**Fig. 1:**
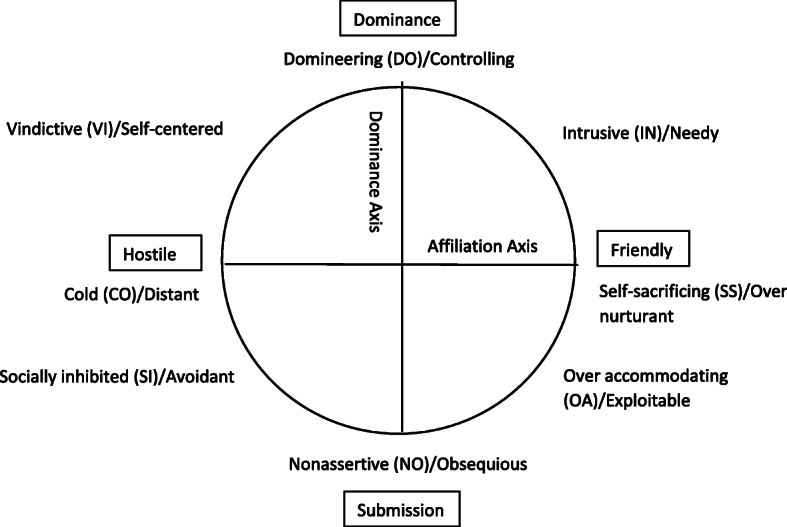
Interpersonal Circumplex

Related studies have shown that overall interpersonal problems were significantly related to IA [31, 32]. Interpersonal problems have, like personality traits, many features, and may be differently involved with PIU. Based on interpersonal circumplex, interpersonal problems are categorized along two intersecting dimensions of affiliation axis (hostile versus friendly), and control axis (dominance versus submission). They produce eight problems: domineering/controlling (DO), vindictive/self-centered (VI), cold/distant (CO), avoidant/socially inhibited (SI), nonassertive/obsequious (NO), exploitable/overly accommodating (OA), over-nurturing/self-sacrificing and intrusive/needy (IN) (Figs. 1), [33]. Only one study, conducted by Seo et al., showed that interpersonal problems and IA significantly correlated [32], especially the types of interpersonal sensitivity, asocial behavior, nonassertiveness, criticize/distrust, and over-nurturing.

Interpersonal problems are related to loneliness, especially the socially inhibited style [34]. An assumption regarding the relationship on PIU was that socially inhibited individuals, also seen as introverted, may use the internet to connect with individuals instead of interacting in real situations [35–37]. A study, using Type D personality, where social inhibition was a part, suggested that introverted individuals were prone to PIU [38]. Moreover, people with hostile (unfriendly) interpersonal style may use Internet as a coping mechanism [39]. A narcissistic person (hostile-dominant) could exhibit a unique approach regarding pathological use of the internet as well [40]. As mentioned before, the contribution to PIU can be complicated [3]. Not one single or specific factor is attributable to PIU. Apart from loneliness and interpersonal problems, Internet use may be influenced by the motivation for Internet use [41].

As Internet becomes ubiquitous in world society and considered as daily life social behavior. Some investigator viewed that PIU can be interpreted as a ordinary shift in these young people to use for entertainment or communication in everyday life rather than pathological behavior [41]. Because of that motivation to use Internet should be included in building a model to explain the PIU. In general, Internet use is related to motivation for enjoyment, entertainment, pleasure seeking [42], high sensation seeking [43], thrill and excitement [44], relatedness, competence, and autonomy [45–47]. Motivation is also influenced by individual’s personality or interpersonal style. For example, antisocial person may use Internet to accomplish illegal activities, to bully or cheat others, and to do illegal gambling [48]. In other occasions, motivation for studying should be considered especially when investigating PIU among medical students [49].

Another motivation from “compensatory Internet use theory” is to use Internet to fulfill unmet real-life, is also related to PIU [2, 22, 41]. This type of motivation is associated with individual’s personality and interpersonal style. For example, if real life of a lonely individual is a lack of social stimulation, he or she may have a motivation to go online to socialize in online game or a social networking site, whereas some avoidant person may have a motivation for escapism by excessively playing online gaming, which ended up being problematic [50, 51]. Internet may be used as a secure place to express hostility [52], or for a displacement [32, 53].

Several studies have shown the role of motivation for Internet use either as a mediator or a moderator. For example, motivation for social interaction mediated the relationship between personality and PIU [4, 54]. Motivation for escapism was found to be a mediator for the relationship between stress and excessive online gaming [41]. Motivation for escapism was also revealed to have moderating effect on the relationship between loneliness and negative outcomes [55].

Based on those aforementioned theories and research evidence, to understand more on PIU, hypothesis to be tested for psychosocial problems as loneliness on PIU, interpersonal problems and motivation for use should be included. However, how each type of interpersonal problem influenced loneliness, motivation of the Internet use and PIU remains little known. The present study aimed to test the relationships among loneliness, a variety of interpersonal problems, motivation for Internet use, and PIU symptoms. The proposed models constituted the interpersonal problems and motivation for Internet use as the mediators for the relationship between loneliness and PIU. Each of the eight subscales of interpersonal problems was examined separately within the prospective models. We hypothesized that all interpersonal subscales would constitute significant mediators but differ in magnitude for the indirect effect of loneliness concerning PIU. As we believed that motivation to use the Internet may be influenced by specific types of interpersonal problems, we tested using a serial mediation model in which each motivation was included as the second mediator. We hypothesized that a significant indirect effect may help understand these specific relationships among the four variables at a deeper level. No prior evidence is available, as to whether motivation for Internet use would produce an interaction effect with some specific type of interpersonal problem concerning PIU. Therefore, we investigated the possibility of moderation effects using an exploratory analysis of moderated-mediation models. Further, we hypothesized that some interpersonal problems and motivations for Internet use may be discovered from its moderating effect on PIU.

## Methods

### Participants and procedures

This study was conducted at the Faculty of Medicine, Chiang Mai University, Chiang Mai, Thailand in 2016. Data were collected cross-sectionally from 318 medical students using convenience sampling. All participants comprised medical students in years 1 to 6. Participants were invited by research assistants with the help of the chair of the medical student union. Each participant provided written informed consent to participate in the study. All completed the paper-pencil questionnaires consisting of 1) demographic data, 2) items concerning motivation of internet use, 2) the IA test, 3) the questionnaires assessing loneliness and 4) the questionnaires assessing interpersonal problems.

### Instruments

1. Questionnaires were used to investigate motivation for Internet use. The first

questionnaire consisted of 24 items concerning respondent’s motivation of internet use, asking how much the respondents agreed to respective objectives using a 5-point scale, ranging from 1 (strongly disagree) to 5 (strongly agree). Examples of the questionnaires included “you use the internet to avoid your real problems in life”, “you want to be accepted from others”, “you want to free yourself by doing something you cannot do it in your real life” In the study Cronbach’s alpha was 0.74.

2. The internet addiction test (IAT), developed by Young [56], is a 20-item self-report instrument in which respondents rate the tendency for IA using a 5-point scale, ranging from 1 (rarely) to 5 (always). Total score yields only an estimate of the overall severity of IA. Higher scores indicate greater addiction. The Thai version was developed by Wongpakaran N. et al. [57]. In the study Cronbach’s alpha was 0.90.

3. The 6-item Revised UCLA Loneliness Scale (RULS-6) is a short form of the revised UCLA

Loneliness scale [58, 59]. Using this self-report instrument, respondents rate the severity of a wide range of feelings of loneliness using a 4-point scale, ranging from 1 (never) to 4 (often). Higher scores indicate greater feelings of loneliness. The RULS-6 demonstrated strong internal consistency (Cronbach’s alpha = 0.72 to 0.84) [59]. In the study Cronbach’s alpha was 0.79.

4. The inventory of interpersonal problems (IIP-32), developed by Horowitz et al. [60], includes 32 questions comprising 8 different interpersonal problems: DO, VI, CO, SI, NO, OA, self-sacrificing (SS) and IN. It uses a self-report instrument in which respondents rate the severity of a wide range of interpersonal problems using a 5-point scale, ranging from 1 (not at all) to 5 (extremely). Each subscale has four items. Higher scores denote greater interpersonal difficulties. The IIP-32 demonstrated strong internal consistency (Cronbach’s alpha = 0.84) and acceptable test-retest reliability (intraclass correlation coefficient = 0.74). The Thai version demonstrated excellent reliability and validity [61], and the scale performed well with this study’s sample (Cronbach’s alpha = 0.88).

The study procedures were carried out in accordance with the Declaration of Helsinki. The Institutional Review Board of the Faculty of Medicine, Chiang Mai University approved the study. All participants were informed about the study, and all provided informed consent.

### Statistical analysis

Sample size was calculated based on the correlation coefficient from a prior study, namely, r = 0.158. Types I and II were determined at 0.05 and 0.20, respectively. Therefore, the minimum required sample size was 312 [62].

Descriptive analysis was performed using socio-demographic, internet-related and all clinical variables by frequency, percentage, and mean standard deviation. Data reduction method was applied for the questionnaires concerning objectives and activities involving internet use, the principal component of analysis. Bivariate correlations between motivation for internet use, loneliness, eight subscales of interpersonal problems and PIU were performed. Analyses were conducted using SPSS, Version 22.

We treated loneliness, eight subscales of interpersonal problems and IA as latent variables. Each of the eight interpersonal subscales was represented in the model by its four observed variables. For the structural equation model, three parcels for the IAT were created. We created parcels for the AIT variables according to loading coefficients by determining them to be parcels 1, 2 and 3 according to loading coefficients. Before testing the structural model, we tested the measurement model and assessed the parcel and subscale loadings on latent constructs. The model fit was assessed using standard χ2 fit statistics, the comparative fit index (CFI), the Tucker-Lewis Index (TLI) and the root mean square error of approximation (RMSEA). A CFI and TLI greater than 0.95 and a RMSEA less than 0.08 indicated a good model fit [63]. Because χ2 statistics is sensitive to sample size, we used the ratio of χ2 to degrees of freedom (χ2/df) to assess model fit. A (χ2/df) ratio of less than 3 indicated an acceptable model fit. Modifications to the initial hypotheses were performed to calculate the modification indices. When the proposed modifications were considered acceptable from a theoretical viewpoint, a new model was elaborated and analyzed.

**Fig. 2:**
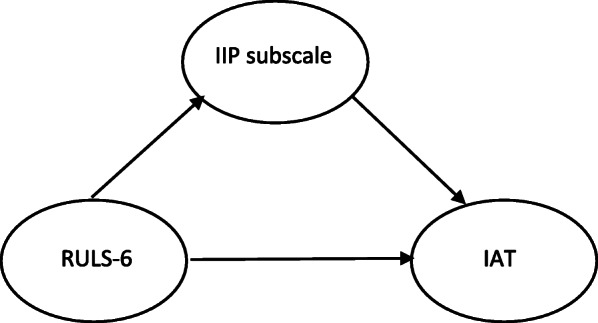
Mediation analysis with structural equation modeling Fig. 1. Structural equation model for mediation (IIP as a latent mediator). IIP = inventory of interpersonal problems, RULS-6 = 6-item revised UCLA loneliness scale, IAT = internet addiction test

Tests of mediation were examined using two steps. In the mediation model, we used latent variables of IIP, RULS-6 and IAT for the first model (Fig. 2), then tested each IIP subscale mediator. Bootstrapping denotes repeated sampling from the data set and estimates the indirect effect in each resampled data set. It provides a more reliable confidence interval (CI) for mediation effect under most conditions, so this method was used to ensure the mediation effect. The bias-corrected and accelerated 95% CI (BCa 95% CI) of the medication effect method was used, and significance effect was determined when the estimated coefficients excluded 0. The bootstrap estimate employed in the present study was based upon 5000 bootstrap samples [64], and all path coefficients were reported in standardized format. AMOS, Version 18 was used for this analysis.

**Fig. 3:**
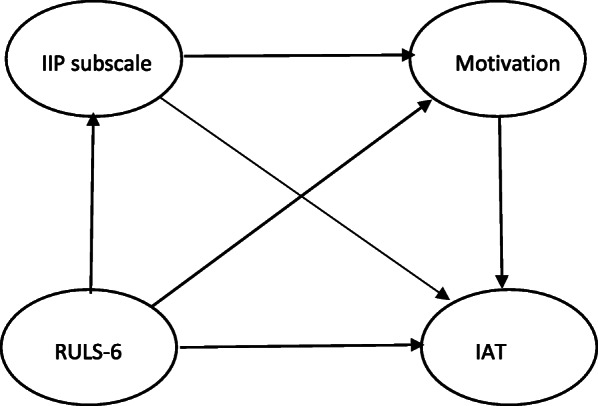
Structural equation model for serial mediation (two latent mediators). IIP = inventory of interpersonal problems, RULS-6 = 6-item revised UCLA loneliness scale, IAT = internet addiction test

**Fig. 4:**
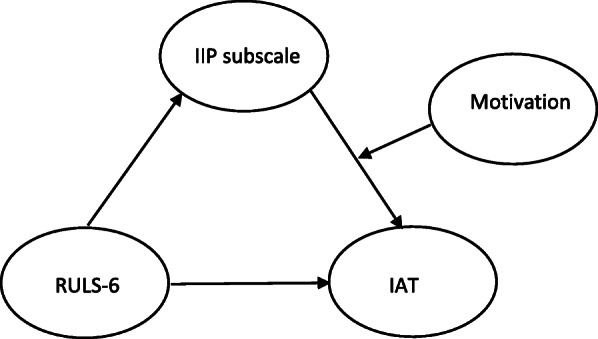
Structural equation model for latent moderated mediation. IIP = inventory of interpersonal problems, RULS-6 = 6-item revised UCLA loneliness scale, IAT = internet addiction test

Serial mediation and moderated mediation were proposed by adding motivation for internet use in the mediation model. Motivation was treated as the second mediator (serial mediation model) or as the moderator of IIP subscale (moderated mediation model). Given that we proposed a serial mediation model, we constructed possible models in which eight interpersonal problems precedes motivation of internet use to determine the importance of variable order (Fig. 3). At the same token, we proposed a moderated mediation, by which each interpersonal problem was moderated by the specific motivation of internet use (Fig. 4). Because latent variables were used instead of observed variables, the latent mediation structural equation model was applied. The percent of variances explained the IAT score that was calculated using R square change to compare between models. Sample size estimation required the desired statistical power level of 0.8, anticipated effect size of 0.3, number of latent variables of 4, and number of observed variables of 18 (including age and sex) totaled 137 at minimum while this study used 324 [65]. Mplus, Version 8.5 [66] with the XWITH command using full information maximum likelihood with robust standard errors was used to assess the latent moderated structural equations model.

## Results

Table 1 shows the participants’ sociodemographic, motivation of internet use, and interpersonal problems. The average time using the internet daily was 4.9 h (SD = 2.7), min-max = 1–20, median = 4.0 h. The PIU level according to Young’s score was 119 (36.7%), comprising mild, 30.9%, moderate, 5.2%, and severe, 0.6% (Table 1). Principal component analysis reduced 24 motivation types of internet use to eight factors, including 1) negative intention, i.e., avoiding real life problems, creating an imaginary self and giving negative feedback or making disparaging remarks, **and** c**onfronting without negative social consequences); 2) being** accepted, i.e., belonging to a group, being recognized and acceptance/praise, 3) taking pleasure, i.e., checking other’s status, recreation and leisure, 4) working, i.e., web board, writing blog/online diary, online banking/business online and shopping/online auction; 5) entertainment, i.e., movies/music, downloading movies, music and YouTube; 6) social connection, i.e., chat and Facebook/Twitter; 7) studying, i.e., Google search, email and e-learning and 8) indulgence, i.e., online games and gambling (see Table S1 for the results of principal component analysis).

**Table 1:** Mean and SD of sociodemographic, motivation of internet use, and interpersonal problems

	Minimum	Maximum	Mean	Std. Deviation
Age	18	24	20.85	1.80
internet addiction total score	5	85	28.18	12.73
RULS-6	6	24	12.36	3.54
***IIP subscale***				
Domineering	0	12	4.49	2.74
Vindictive	0	11	3.68	2.44
Cold	0	12	4.28	2.88
Socially inhibited	0	15	4.66	3.02
Nonassertive	0	12	6.44	2.53
Overly accommodating	0	14	6.59	2.71
Self-sacrificing	0	13	6.24	2.78
Intrusive	0	14	4.75	2.75
IIP total score	2	83	41.29	13.52
***Motivation of internet use***				
Negative intention	4	16	6.06	2.41
Being accepted	3	14	6.92	2.59
Taking pleasure	6	15	12.11	1.87
Working	4	16	6.06	2.41
Entertainment	3	14	6.92	2.59
Social connection	6	15	12.11	1.87
Studying	4	15	6.83	2.37
Indulgence	4	15	11.06	2.19

Note: IIP = the inventory of interpersonal problems, RULS-6 = 6-item revised UCLA loneliness scale

The RULS-6 score and all subscales of interpersonal problems had significant relationship with IAT score (all *p* < .01), except for IN subscale. The DO subscale did not exhibit a significant relationship with NO and OA subscales, whereas VI did not show a significant relationship with self-sacrifice and IN subscales, which overall was compatible with the interpersonal circumplex in that they are on the opposite character. All these subscales were treated as an independent trait for the structural equation model. Age significantly correlated with working online, social connection and indulgence, whereas sex significantly correlated among entertaining, studying and indulgence. Different interpersonal subscales were associated with different internet activities. For example, DO and IN subscales were significantly related to negative intention, **being** accepted and working. The remaining details are shown in Table S2.

### Testing for mediation effect

All measurement models were tested separately, and all models were found to indicate good fit statistics. Table 2 shows that both the direct and the indirect effects of the eight IIP subscales on IAT were significant, except for IN and CO, which had no significant indirect effect. Fit statistics showed that all models fit the sample well.

**Table 2:** Total, direct, and indirect effects of RULS-6 and IIP subscale on IAT controlling for age and sex

Mediator (IIP subscale)	Total effect RULS-6 ➔ IAT	Direct effect of RULS-6 ➔ mediator(a)	Direct effect of RULS-6 ➔ IAT (c’)	Direct effect of mediator ➔ IAT(b)	Indirect effect (a* b)	χ^2^	df	χ^2^ /df	CFI	TLI	RMSEA
DO	.32***	.32***	.24**	.23**	.08**	82.01	70	1.17	.99	.99	.02(.00–.04)
VI	.31***	.41***	.20**	.26**	.11**	130.12	71	1.83	.96	.95	.05(.04–.06)
CO	.31***	.61***	.24*	.13	.08	131.83	71	1.86	.96	.95	.05(.04–.07)
SI	.33***	.66***	.17	.24*	.15*	130.41	85	1.53	.97	.96	.04(.03–.05)
NO	.32***	.41***	.26***	.16*	.07*	114.72	68	1.69	.96	.95	.04(.03–.06)
OA	.32***	.47***	.21*	.23*	.11*	81.90	67	1.22	.99	.98	.03(.00–.04)
SS	.32***	.39***	.19*	.32***	.12***	101.01	68	1.48	.98	.97	.04(.02–.05)
IN	.32***	.06	.31***	.26**	.01	119.11	67	1.78	.96	.95	.05(.03–.06)

Note: IIP = inventory of interpersonal problems, DO = domineering/controlling, VI = vindictive, CO = cold, SI = socially inhibited, NO = nonassertive, OA = overly accommodating, SS = self-sacrificing, IN = intrusive, χ^2^ = Chi-square, df = degree of freedom, CFI = comparative fit index, TLI = Tucker-Lewis Index, RMSEA = Root-mean-square error of approximation, RULS-6 = 6-item revised UCLA loneliness scale, IAT = internet addiction test

***p < .001, **p < .01,*p < .05

Among all eight mediators, SI appeared to exhibit the highest effect size of mediation effect because its direct effect on IAT (c’) was reduced to nonsignificant (*p* = .08).

The results showed that six dimensions of IIP served as mediators, from partial or full, for loneliness concerning IA, although they differed in style or were even opposite such as DO and NO. Subsequently, each of the motivation types for internet use was included in the mediation models creating serial mediation analysis (Fig. 3), and each motivation of the Internet was used a moderator of the IIP subscale to create moderated mediation analysis (Fig. 4). Altogether, 128 (64 serial mediation + 64 moderated mediation) models were tested individually.

Among all 64 serial mediation, SEMs, only 12 models were accepted, in which Mediator 1 were DO, CO, VI, SI, OA, IN. Negative intention and working had a significant indirect effect on IAT (β = 0.057 to − 0.123, *p* < .05). None of any significant indirect models was found in SS and NO (Table 3), and the fit statistics revealed all models fit the sample well.

**Table 3:** Results of the serial mediation models controlling for age and sex (only significant indirect effects)

Mediator (M1)	Mediator (M2)	Indirect effect of RULS-6➔ IAT through M1and M2: β (SE)	*p*-value
DO	Being accepted	.11(.04)	.01
	Negative intention	.40(.07)	.00
	Working online	.07(.04)	.03
CO	Being accepted	.22(.07)	.00
	Negative intention	.36(.08)	.00
VI	Being accepted	.19(.05)	.00
	Negative intention	.39(.07)	.00
	Working online	.11(.04)	.00
	Studying	.09(.04)	.04
SI	Being accepted	.31(.07)	.00
	Negative intention	.47(.09)	.00
	Working online	.18(.06)	.00
	Studying	.15(.06)	.01
	Indulgence	.18(.08)	.02
	Social connection	.15(.08)	.04
SS	Being accepted	.17(.04)	.00
	Negative intention	.43(.08)	.00
	Working online	.12(.04)	.00
	Studying	.10(.04)	.01
NO	Being accepted	.13(.04)	.00
	Negative intention	.38(.08)	.00
	Working online	.10(.04)	.02
OA	Being accepted	.16(.05)	.00
	Negative intention	.37(.08)	.00
	Working online	.11(.05)	.02
IN	Being accepted	.09(.04)	.03
	Negative intention	.35(.07)	.00

Note:

β = standardized estimate, SE = standard error, DO = domineering, VI = vindictive, CO = cold/distant, SI = socially inhibited, NO = nonassertive, OA = overly accommodating, SS = self-sacrificing, IN = intrusive, RULS-6 = 6-item revised UCLA loneliness scale, IAT = internet addiction test

When latent variables of motivation interacted with the IIP subscale, the motivation of negative intention and being accepted mediated all eight of the IIP subscales. Motivation of working online mediated all interpersonal problems, except for CO and IN. Notably, IN had no mediation effect but when combined with negative intention, it produced the effect of IN and negative intention. For SI, all activities but social connection became the second mediators and produced a significant indirect effect concerning loneliness to IAT (Table 4).

**Table 4:** Results of the latent moderated mediation controlling for age and sex (only significant total indirect effects)

Mediator (M1)	Moderator (M2)	Interaction effect of M1 and M2 on IAT: β (SE)	*t*	p-value	Total Indirect effect: β (SE)	*t*	p-value
DO	–	–	–	–	–	–	–
VI	Negative intention	.18 (.08)	2.13	.03	.13 (.05)	2.75	.00
CO	–	–	–	–			
SI	Working	.28 (.09)	3.14	.00	.29(.10)	2.87	.00
NO	Working	.29 (.12)	2.49	.01	.23(.09)	2.50	.01
OA	Working	.26 (.08)	3.30	.00	.22(.08)	2.50	.01
SS	Working	.25 (.12)	2.18	.03	.25(.10)	2.53	.01
IN	–	–	–	–	–	–	–

Note: β = standardized estimate, SE = standard error, RULS-6 = 6-item revised UCLA loneliness scale, IAT = internet addiction test

*t* is calculated by β / SE, DO = domineering, VI = vindictive, CO = cold, SI = socially inhibited, NO = nonassertive, OA = overly accommodating, SS = self-sacrificing, IN = intrusive

Table 4 shows significant moderators of motivation and total indirect effects of IIP subscales. DO and IN were not moderated by any motivation. Among all 64 interacted latent variables, only negative intention moderated VI, and working moderated SI, NO, OA and SS. In addition, a significant indirect effect of these IIP subscales was found through the moderators with *t* = 2.75, *p* < 0.01; *t* = 2.87, *p* < 0.01; *t* = 2.50, *p* = 0.01; *t* = 2.50, *p* = 0.01 and *t* = 2.53 *p* = 0.01, respectively.

In terms of R square change, the variance of IAT explained by all variables in the mediation model (one mediator of interpersonal problems) was approximately 13% and increased to approximately 43% for the serial mediation model (two mediators), and to approximately 33% for the moderated mediation model (one mediator of interpersonal problems, one moderator).

## Discussion

This study aimed to examine how loneliness was associated with PIU using interpersonal problems and motivation for Internet use as mediators. It became evident that interpersonal problem served as a mediator for loneliness and PIU. Motivation for Internet use contributed to increased variances explained for PIU, denoting their significant effect on the relationship between loneliness and interpersonal problems, and PIU.

Socially inhibited problem was the strongest mediator among all, showing full mediation, denoting that the association between loneliness and PIU might be nonsignificant when socially inhibited interpersonal problems was removed. This finding was observed by Horowitz and French [67] in that lonely individuals consistently reported problems of inhibited sociability. The remaining interpersonal problems produced a mediation effect, but exhibited a partial effect, except for the IN, which had no mediation effect.

As hypothesized, specific motivation for Internet use related to some interpersonal problems and resulted in an effect on PIU. The motivation of being accepted and of negative intention were found to be the second mediator for all models, regardless of interpersonal problems. In a word, regardless of the type of interpersonal problems an individual has, desire to be accepted and to receive social support is common [68–70]. On the contrary, negative intention including defamatory remarks, and bullying others was associated with all types of interpersonal problems, not only negative ones as found among individuals with dark side personality [52].

Negative intention may not be that surprising when it occurs in the VI subscale, a negative interpersonal style, but what could be the explanation considering the friendly style such as OA? Recall that interpersonal problems do not constitute a cognitive style [71] as even the friendly interpersonal style could express negative thoughts. Investigators have revealed that OA significantly correlated with fantasy proneness, irresponsibility and negative affect, which was endorsed in the negative intention factor found in the present study [72]. In other words, participants feeling lonely may be likely to become internet addicts when they had negative thoughts and intentions (feelings that bring out the worst in them), because the internet could constitute the network where they can safely express their abuse, which is difficult to do so in real life. As endorsed by Kircaburun & Griffiths, the Internet is a safe place to hide their real self and invoke or express their negative intentions to others [52].

Notably, SI was the only IIP subscale relating to various types of motivation either positive or negative. A motivation for social connection such as chatting, using Facebook or Twitter, and searching the internet may not differentiate addicted individuals from normal users, and did not affect IA score [73]. However, SI became one of the important variables associated between loneliness and PIU. It may reflect that people who are socially inhibited tend to access the Internet much more with a variety of motivations, particular in escapism using online gaming as found in the related study [74].

Another point is that IN was not a mediator in the first place but could become one when the motivation variables were added. This highlights the importance of unobserved variables like motivation to be accepted or to express negative feeling on PIU that may prevail over any particular type of interpersonal problem. Interestingly, motivation for working online became both a mediator and a moderator in some types of IIP. Regarding mediation effect, it may be unsurprising that working served as an additional effect on the model but in the moderating model, working become a moderator only with submissive style of IIP, i.e., SI, NO, OA and SS, meaning that these IIP subscales differentially predicted IA as a function of working online. If the graph were to be plotted between IIP subscale and IA based on the high and low level of working online, it would illustrate that at medium to high levels of working online, the magnitude of correlations (slope) between them was significantly greater than at lower level (sharper slope). This could provide us the insight that when individuals with a submissive character feel lonely and maintain a high level of working online, they tend to become excessive Internet users, and then would be considered PIU. Another moderation effect was found in VI and negative intention, indicating that the higher the level of negative intention, the higher predictive level of VI concerning IA could be observed. This is, however, not beyond expectation.

Overall, we have attempted to determine what makes each pair of IIP subscales and loneliness differ concerning PIU. They all involve and are also determined by the motivation of Internet use. Although motivation of the users sheds some light on the link between loneliness, interpersonal problem and IA, more models remain untested, which might yield a better fit and understanding of clinical data, for example, moderation between motivation and loneliness concerning IIP and moderation between motivation and loneliness concerning IAT. Further investigation is warranted.

The main implication for future research is to examine the potential causes of excessive Internet use or PIU that may be involved in the motivation for Internet use. These findings underline that a view of excessive Internet use may be explained as a coping strategy based on an existing interpersonal style, rather than only as a mental disorder, as suggested by many researchers. To study PIU, interpersonal problems as well as motivation for Internet use should be incorporated with the researcher’s interested independent (predicting) variables. We believe this strategy would provide more insight from those newly proposed models.

## Strengths and limitations

This may constitute complicated models’ analysis regarding a mediation model of loneliness and interpersonal problems concerning PIU. Moreover, including motivation types in the structural model was accomplished for the first time to explain the differences between the effects each interpersonal problem has on IAT score. For this the summary findings presented were derived from overall extensive analyses of 136 models. However, our study encountered some limitations. First, this study was confined to a medical student population and might not be generalized to a conventional population. Second, depression was excluded, which may be linked to loneliness (or interpersonal problems). This may have made it difficult to conclude that interpersonal problems were the only mediator for loneliness although depression might have also contributed. Moreover, a separate analysis based on type of PIU may be useful to obtain more insights concerning the varied nature of addiction among users. Lastly, this study used self-reported measures, so bias might have occurred due to social desirability.

## Conclusion

This research highlighted the importance of interpersonal problems and motivation among PIUs who experience loneliness. Our findings revealed various interpersonal problems, except for the cold style, that partially mediated the relationship between loneliness and PIU. Motivation for being accepted and negative intention were the most common as the second mediator for all types of interpersonal problems. Further, most of the motivation mediated the relationship between social inhibition and Internet addiction in the serial mediation models. Among all motivations, only negative intention showed a moderating effect of vindictive style, whereas motivation for working revealed a moderating effect of all the submissive styles of the interpersonal problems. It seems including the interpersonal problems and motivation for Internet use is important as these are likely to have a stronger impact on the outcomes.

## Supplementary Information

**Additional file 1.**

**Additional file 2.**

## Data Availability

The datasets used and/or analyzed during the current study are available from the corresponding author on reasonable request.

## Abbreviations

IIP: Inventory of Interpersonal problems
RULS-6: The 6-item of the revised University of California Los Angeles Loneliness Scale
IAT: Internet addiction test
DO: Domineering
VI: Vindictive
CO: Cold
SI: Socially inhibited
NO: Nonassertive
OA: Overly accommodating
SS: Self-sacrificing
IN: Intrusive
