# Supplementary material for: Loneliness and problematic internet use: testing the role of interpersonal problems and motivation for internet use
Source: BMC Psychiatry. 2021 Sep 10;21:447. doi: 10.1186/s12888-021-03457-y (PMC8431860; doi:10.1186/s12888-021-03457-y)
Supplement: Supplementary file 1 — Additional file 1. [file 12888_2021_3457_MOESM1_ESM.docx]

| Table S1. The results of principal component analysis of motivation for internet use | | | | | | | | |
| --- | --- | --- | --- | --- | --- | --- | --- | --- |
|  | Component | | | | | | | |
| Motivation | 1 | 2 | 3 | 4 | 5 | 6 | 7 | 8 |
| Avoid real life problems | **.742** |  |  |  |  |  |  |  |
| Create imaginary self | **.741** |  |  | .130 |  |  |  |  |
| Making negative feedback or disparaging remarks | **.663** | .167 |  | .149 |  |  |  | .149 |
| Confronting without negative social consequences | **.454** |  |  | .357 | -.210 | .205 | .203 |  |
| Being recognized | .279 | **.729** |  | .116 |  |  |  | -.227 |
| Accept/ praise | .395 | **.686** |  | .100 |  |  |  |  |
| Chatting | -.150 | **.640** | .193 |  | .187 | .105 | .204 | .187 |
| Social networking (facebook/twitter) | -.126 | **.507** | .316 |  | .140 | .288 | .242 | .239 |
| Belonging to a group | .389 | **.416** | .106 | .185 |  | .213 | -.196 | -.351 |
| Movies/music |  |  | **.759** |  | -.225 |  | .120 | -.108 |
| Youtube |  | .154 | **.704** |  | .149 |  |  |  |
| Downloading (movies/ music etc.) |  | .147 | **.685** | .121 | .201 |  | -.110 | .150 |
| Leisure |  |  | **.413** |  |  | .381 | .165 |  |
| Blogging/ online diary | .165 |  | .106 | **.696** |  | -.182 |  | -.226 |
| Online banking/ business online | .154 |  |  | **.650** | .241 |  | .369 |  |
| Online forum participation (Web board) |  | .102 | .109 | **.622** |  | .234 |  | .162 |
| Online gambling |  | .244 | -.208 | **.507** | -.164 |  | -.163 | .215 |
| Searching for information (e.g., google) |  |  | .189 |  | **.710** | .132 |  |  |
| Reading E-learning |  | .118 |  | .236 | **.663** | -.131 | -.306 |  |
| Emailing |  |  | -.136 | .130 | **.633** | .121 | .467 | -.113 |
| Recreation | .159 |  |  |  |  | **.780** |  |  |
| Checking other's status | -.143 | .174 | .261 | .128 |  | **.682** | -.221 | -.154 |
| Shopping/auction |  | .232 | .152 | .169 |  |  | **.732** |  |
| Online games | .207 |  |  |  |  |  |  | **.829** |
