# Supplementary material for: Loneliness and problematic internet use: testing the role of interpersonal problems and motivation for internet use
Source: BMC Psychiatry. 2021 Sep 10;21:447. doi: 10.1186/s12888-021-03457-y (PMC8431860; doi:10.1186/s12888-021-03457-y)
Supplement: Supplementary file 2 — Additional file 2. [file 12888_2021_3457_MOESM2_ESM.docx]

| Table S1. Zero-order correlation between variables | | | | | | | | | | | | | | | | | | | | |
| --- | --- | --- | --- | --- | --- | --- | --- | --- | --- | --- | --- | --- | --- | --- | --- | --- | --- | --- | --- | --- |
|  | 1 | 2 | 3 | 4 | 5 | 6 | 7 | 8 | 9 | 10 | 11 | 12 | 13 | 14 | 15 | 16 | 17 | 18 | 19 | 20 |
| AGE |  |  |  |  |  |  |  |  |  |  |  |  |  |  |  |  |  |  |  |  |
| SEX | .08 | - |  |  |  |  |  |  |  |  |  |  |  |  |  |  |  |  |  |  |
| RULS-6 | -.04 | -.01 | - |  |  |  |  |  |  |  |  |  |  |  |  |  |  |  |  |  |
| IAT | -.04 | .07 | .29^**^ | - |  |  |  |  |  |  |  |  |  |  |  |  |  |  |  |  |
| Do | -.03 | -.05 | .24^**^ | .25^**^ | - |  |  |  |  |  |  |  |  |  |  |  |  |  |  |  |
| VI | .03 | -.04 | .31^**^ | .27^**^ | .27^**^ | - |  |  |  |  |  |  |  |  |  |  |  |  |  |  |
| cold | -.08 | -.08 | .48^**^ | .25^**^ | .17^**^ | .59^**^ | - |  |  |  |  |  |  |  |  |  |  |  |  |  |
| SI | -.04 | -.14^*^ | .54^**^ | .29^**^ | .15^**^ | .54^**^ | .69^**^ | - |  |  |  |  |  |  |  |  |  |  |  |  |
| NA | -.05 | -.03 | .30^**^ | .22^**^ | .07 | .34^**^ | .45^**^ | .46^**^ | - |  |  |  |  |  |  |  |  |  |  |  |
| OA | -.05 | -.04 | .35^**^ | .20^**^ | .02 | .19^**^ | .41^**^ | .41^**^ | .64^**^ | - |  |  |  |  |  |  |  |  |  |  |
| SS | -.07 | .01 | .33^**^ | .31^**^ | .28^**^ | .03 | .22^**^ | .22^**^ | .34^**^ | .60^**^ | - |  |  |  |  |  |  |  |  |  |
| IN | .05 | .10 | .11 | .26^**^ | .52^**^ | .10 | .02 | -.02 | .20^**^ | .18^**^ | .42^**^ | - |  |  |  |  |  |  |  |  |
| NI | -.04 | .01 | .38^**^ | .41^**^ | .18^**^ | .22^**^ | .34^**^ | .32^**^ | .20^**^ | .27^**^ | .23^**^ | .14^*^ | - |  |  |  |  |  |  |  |
| BA | -.01 | .09 | -.02 | .23^**^ | .11^*^ | -.09 | -.16^**^ | -.18^**^ | -.04 | -.07 | .04 | .21^**^ | .00 | - |  |  |  |  |  |  |
| TP | -.03 | .03 | -.09 | .24^**^ | .08 | -.02 | -.04 | .02 | -.02 | -.02 | -.00 | .06 | .00 | .01 | - |  |  |  |  |  |
| WO | .17^**^ | .07 | .01 | .16^**^ | .24^**^ | .04 | -.03 | .00 | -.05 | -.03 | .08 | .14^*^ | .25^**^ | .19^**^ | -.01 | - |  |  |  |  |
| EN | -.09 | .21^**^ | -.04 | .25^**^ | .03 | -.03 | -.06 | -.03 | .01 | .01 | .06 | .01 | -.07 | .15^**^ | .23^**^ | -.00 | - |  |  |  |
| SC | .12^*^ | .11 | -.11^*^ | .21^**^ | .12^*^ | -.07 | -.09 | -.17^**^ | -.08 | -.10 | .01 | .16^**^ | .00 | .24^**^ | .27^**^ | -.00 | .014 | - |  |  |
| ST | .07 | -.15^**^ | .01 | -.02 | -.05 | -.01 | -.02 | -.06 | -.02 | .06 | -.03 | -.06 | -.08 | .01 | .04 | .01 | -.00 | .00 | - |  |
| IND | -.18^**^ | -.34^**^ | .03 | .16^**^ | .01 | .02 | .04 | .05 | .03 | .04 | .01 | -.00 | .15^**^ | .05 | -.02 | .01 | .11 | -.01 | -.01 | - |
| **Note: IIP = the inventory of interpersonal problems,** RULS-6 = 6-item revised UCLA loneliness scale, IAT = internet addiction test, RULS-6 = 6-item revised UCLA loneliness scale, DO = domineering, VI = vindictive, CO = cold, SI = socially inhibited, NO = nonassertive, OA = overly accommodating, SS = self-sacrificing, IN = intrusive, IN=Negative intention, BA=Being accepted, TP=Taking pleasure, WO=Working, EN=Entertainment, SO=Social connection, ST=Studying, IND=Indulgence | | | | | | | | | | | | | | | | | | | | |
